# Selection of and Response to Physical Activity–Based Social Comparisons in a Digital Environment: Series of Daily Assessment Studies

**DOI:** 10.2196/41239

**Published:** 2023-02-27

**Authors:** Danielle Arigo, Robert C Gray, Diane H Dallal, Jennifer Villareale, Jichen Zhu

**Affiliations:** 1 Department of Psychology Rowan University Glassboro, NJ United States; 2 Department of Family Medicine Rowan-Virtua School of Osteopathic Medicine Stratford, NJ United States; 3 Department of Digital Media Drexel University Philadelphia, PA United States; 4 Department of Psychological and Brain Sciences Drexel University Philadelphia, PA United States; 5 Weight, Eating, and Lifestyle Science Center Drexel University Philadelphia, PA United States; 6 Department of Digital Design IT University of Copenhagen Copenhagen Denmark

**Keywords:** social comparison, physical activity, motivation, web platform, selection, exercise, fitness, mobile phone

## Abstract

**Background:**

Innovative approaches are needed to understand barriers to and facilitators of physical activity among insufficiently active adults. Although social comparison processes (ie, self-evaluations relative to others) are often used to motivate physical activity in digital environments, user preferences and responses to comparison information are poorly understood.

**Objective:**

We used an iterative approach to better understand users’ selection of comparison targets, how they interacted with their selected targets, and how they responded to these targets.

**Methods:**

Across 3 studies, different samples of insufficiently active college students used the Fitbit system (Fitbit LLC) to track their steps per day as well as a separate, adaptive web platform each day for 7 to 9 days (N=112). The adaptive platform was designed with different layouts for each study; each allowed participants to select their preferred comparison target from various sets of options, view the desired amount of information about their selected target, and rate their physical activity motivation before and after viewing information about their selected target. Targets were presented as achieving physical activity at various levels below and above their own, which were accessed via the Fitbit system each day. We examined the types of comparison target selections, time spent viewing and number of elements viewed for each type of target, and day-level associations between comparison selections and physical activity outcomes (motivation and behavior).

**Results:**

Study 1 (n=5) demonstrated that the new web platform could be used as intended and that participants’ interactions with the platform (ie, the type of target selected, the time spent viewing the selected target’s profile, and the number of profile elements viewed) varied across the days. Studies 2 (n=53) and 3 (n=54) replicated these findings; in both studies, age was positively associated with time spent viewing the selected target’s profile and the number of profile elements viewed. Across all studies, upward targets (who had more steps per day than the participant) were selected more often than downward targets (who had fewer steps per day than the participant), although only a subset of either type of target selection was associated with benefits for physical activity motivation or behavior.

**Conclusions:**

Capturing physical activity–based social comparison preferences is feasible in an adaptive digital environment, and day-to-day differences in preferences for social comparison targets are associated with day-to-day changes in physical activity motivation and behavior. Findings show that participants only sometimes focus on the comparison opportunities that support their physical activity motivation or behavior, which helps explain previous, equivocal findings regarding the benefits of physical activity–based comparisons. Additional investigation of day-level determinants of comparison selections and responses is needed to fully understand how best to harness comparison processes in digital tools to promote physical activity.

## Introduction

### Background

Engaging in regular physical activity (PA) has wide-ranging and meaningful benefits for physical and mental health [1-3]. Although activity of moderate to vigorous intensity confers unique cardiovascular protection [4], lighter-intensity activity is linked to positive outcomes and is recommended to promote health [5,6]. Conversely, physical inactivity is a key contributor to many of the leading causes of death in the United States and worldwide, including cardiovascular disease and cancer [7-9]. Identifying determinants of PA engagement has been a research priority for several decades and has informed a myriad of prevention and intervention efforts [10]. However, despite these efforts, adults in the United States rarely engage in sufficient PA to protect their health; recent estimates indicate that only 50% meet recommended levels of PA [11], although estimates vary by calculation approach [12]. Consequently, there is a clear need for work that can offer additional insights into PA barriers and facilitators—particularly those that could inform PA promotion efforts on a large scale.

Digital tools such as web platforms and mobile apps show promise for maximizing accessibility to PA resources as they are available for use as needed and can respond to varying contexts in daily life. Specifically, these tools can harness the power of the social environment to support PA by connecting individuals with other users without requiring synchronous interaction [13]. For example, social comparison processes can be activated by sharing PA data between users as captured by a wearable monitor [14]. Exposure to others’ PA behavior allows users to evaluate their own PA relative to that of others [15] using features such as leaderboards and competitive challenges [16,17]. Upward comparison, via exposure to someone doing better with PA (eg, with more steps per day), can inspire the comparer to reach the upward target’s level and provide guidance for how to achieve a similar outcome [18]. Downward comparison, via exposure to someone doing worse with PA (eg, with fewer steps per day), can prompt the comparer to avoid becoming like the downward target to maintain their status [19,20]. Social comparison is expected to work in these ways to motivate users to maintain or increase their PA [21,22].

As a result, features of digital PA tools that activate social comparison processes are popular and have received considerable attention [14,23]. Literature in this area shows some evidence that social comparisons affect PA motivation and behavior (via digital tools and more broadly [24-26]). For instance, direct access to information about others’ PA behavior results in attending more group exercise classes than access to discussions with others about PA (to facilitate social support [27]). However, the effects of comparisons in both upward and downward directions on PA outcomes are heterogeneous and poorly understood. Some people experience *decreased* PA motivation or behavior in response to social comparisons, including those that are self-selected from a range of options [27-29].

Furthermore, responses to comparisons of PA (with respect to motivation and behavior) are not necessarily consistent for the same person across time; a person may respond positively at some times and negatively at others depending on the daily context [30]. In addition to the direction of a comparison (upward vs downward), a feature that may affect a comparison’s proximal influence on PA outcomes is its scale, or the relative distance between the comparer and target. Comparisons to others doing just a little bit better or worse than the self may have the biggest impact as the target’s outcome seems achievable (upward) or imminent (downward) and the comparer is motivated to improve or maintain their status [15,31,32]. In contrast, comparisons to others who are doing much better or worse may be demotivating as the target’s outcome seems unattainable (upward) or unlikely (downward).

Despite the ubiquity of social comparison features in digital tools to promote PA, the optimal approach to activating comparison processes in a digital environment is not clear. Allowing users to select their preferred comparison target appears to be more effective for promoting PA than restricting exposure to a single (nonpreferred) target [33], and many digital comparison opportunities allow the user to select or focus on a subset of targets from a range of options (eg, leaderboards). However, as noted, even self-selection often results in negative responses. Specifically, there is a current need for additional insights into users’ comparison selections, their interactions with these selections, and the extent to which users respond positively (vs negatively) to their selections in a digital PA environment.

### Aims of This Study

Given the availability of digital features that activate social comparison processes to promote PA and the equivocal nature of evidence in this area, there is a need for an improved understanding of PA-based comparison selections and responses in a digital environment. Additional information in this domain could elucidate the nature of PA-based comparison processes and help identify the comparisons that are associated with benefits for PA outcomes (vs harms). The aims of this study were to describe PA-based comparison selections (direction and scale) and examine day-level associations between comparison selections and PA outcomes (motivation and behavior), both overall and for within-person differences across days. To achieve these aims, we used data from an existing 3-study series that allowed participants to select a PA-based comparison target from different sets of options with respect to direction and scale. PA motivation was assessed both before and after comparison exposure each day, and PA behavior was captured in steps per day using the Fitbit platform (Fitbit LLC).

## Methods

### Study Series Overview

As part of a larger series of studies to investigate the potential for personalizing social comparison opportunities in the context of a social exergame [34-37], participants in each study completed 7 to 28 total days of data collection. In studies 2 and 3, the first 9 days constituted an exploratory period during which all participants selected from various sets of comparison options; the following days introduced a personalized experimental manipulation for half of the participants based on random assignment. This report describes a set of secondary analyses that examine comparison selections, interactions with these selections via a web platform, and associated consequences for PA motivation and behavior during only the initial 7- or 9-day exploratory period in each study.

### Recruitment and Eligibility

#### Consistent Components Across Studies

Across studies, participants were recruited from the Drexel University undergraduate student participant pool using both in-class recruiting and a web-based study scheduling platform (Sona Systems). Students were eligible to participate if they were aged ≥18 years, had daily access to a desktop or laptop computer, self-reported that PA was important to them, and had access to a Fitbit account or were willing to create one. Use of either a Fitbit wearable device or the Fitbit smartphone app was acceptable. Students were excluded if they had a medical condition that limited their ability to engage in moderate- or vigorous-intensity PA or were under medical advisement to avoid moderate or vigorous PA.

#### Participants—Study 1

Of the 11 undergraduate students who expressed interest in participating, 6 (55%) enrolled in this initial pilot phase. In total, 17% (1/6) of the participants did not complete any days of data collection and were excluded, resulting in a sample of 5 students. The average participant took 4690 (SE 1767.99) steps per day during the study period. All participants were undergraduate students aged ≥18 years; however, further demographic data were not collected during this initial pilot.

#### Participants—Study 2

Through rolling recruitment over the course of 2 months, 119 students expressed interest in participating. Of these 119 students, 66 (55.5%) did not complete the required days of data collection, resulting in 53 (44.5%) participants who enrolled in study 2. The sample comprised 57% (30/53) women and was racially representative of an undergraduate population, with most participants identifying as White (28/53, 53%) or Asian (13/53, 25%; see Table 1 for further demographic information). The average participant took 6376 (SE 351.43) steps per day during the baseline study period.

**Table 1:** Demographic characteristics of each sample (N=112).

Demographics		Study 1^a^ (n=5)	Study 2 (n=53)	Study 3 (n=54)
**Gender, n (%)**				
	Women	—^b^	30 (57)	37 (69)
	Men	—	23 (43)	17 (31)
Age (years), mean (SD; range)		≥18	22.45 (7.40; 18-53)	20.31 (2.93;18-36)
**Race, n (%)**				
	White	—	28 (53)	23 (43)
	Asian	—	13 (25)	22 (41)
	Multiracial	—	5 (9)	4 (7)
	Black	—	4 (8)	2 (4)
	Other	—	2 (4)	2 (4)
	American Indian or Alaska Native	—	1 (2)	0 (0)
	Prefer not to say	—	0 (0)	1 (2)
**Ethnicity, n (%)**				
	Hispanic or Latino	—	3 (6)	7 (13)
	Not Hispanic or Latino	—	49 (92)	47 (87)
	Not reported	—	1 (2)	0 (0)

^a^Demographic data were not collected for study 1.

^b^Not available.

#### Participants—Study 3

Through rolling recruitment over 3 months, 90 students expressed interest in participating. Of these 90 students, 35 (39%) did not complete the required days of data collection, resulting in 54 (60%) participants who enrolled in study 3. Most of the participants were women (37/54, 69%) and the majority of students identified as White (23/54, 43%) or Asian (22/54, 41%; see Table 1 for further details). The average participant took 3609 (SE 339.32) steps per day during the baseline study period.

### Measures

#### Social Comparison Selections

As described in the following sections, participants were asked to select user profiles to view each day from a range of options that represented upward and downward comparisons relative to their own PA behavior. They could select multiple profiles each day to view partial information but could only select 1 profile to view in full. Telemetry built into the web application tracked the participants’ navigation of the web app, including the profiles they viewed (in part or in full), the time spent viewing profiles, and the fields they chose to observe for their full selected profile. Comparison selections were operationally defined with respect to the total number each day, the time spent viewing profiles, the number of profile elements viewed, and the direction and scale of the profile selected for full viewing.

#### Motivation to Exercise

Participants in studies 2 and 3 self-reported their immediate motivation to exercise at the start and end of their participation each day (ie, before and after their comparison selections and exposure). Responses to the following statement—“Overall, I would rate my current motivation to exercise as...”—were rated on a scale from 1 (very low motivation) to 5 (very high motivation) at each time point. This approach to assessing motivation was guided by previous work in this area, including prior work by the investigators [28,38].

#### PA Behavior

To maximize accessibility, activity behavior was defined as total steps per day; steps are a commonly used metric to evaluate PA behavior and are associated with health outcomes [39]. Daily step count totals were measured using data pulled from either a Fitbit wearable device or the Fitbit MobileTrack smartphone app. The app is synced to a participant’s accelerometer on their smartphone, which shows validity for assessing steps across devices and operating systems [40]. Of note, we allowed for heterogeneity in the device used to measure daily steps to enhance the generalizability of findings across individuals with and without the means to purchase a wrist-worn device. This approach has been used in prior work, which shows that Fitbit devices and the MobileTrack app do not generate meaningfully different step estimates [41]. Fitbit step data from the previous day were synced with the study website and then displayed to participants when they logged into the study platform each day.

### Procedures

After completing a web-based screening survey to determine eligibility, eligible individuals provided electronic informed consent and were then directed to a second web page where they completed a battery of global self-report questionnaires (not included in this report). Participants were then given a username and log-in for the daily web-based activity, where on first log-in, they were directed to authenticate a Fitbit account with our web platform so that daily steps could be retrieved. Starting the following day (which allowed for the sign-up day’s steps to be used in the first session), the user was introduced to the relevant activities described in the following sections. Users were asked to log in and complete a session once per day; the time of day was not specified.

Upon log-in, the web server queried the user’s steps for the previous day via the Fitbit application programming interface (API). If it was detected via the API call that Fitbit did not yet have a full account of the previous day’s steps, the web application directed the participant to open the Fitbit app on their mobile device to prompt a data upload. Of note for study 2, there was a short period during data collection (3 days) in which the Fitbit server was not properly syncing with the study website. As a result, participants’ steps displayed upon logging in represented steps from the last successful sync rather than from the previous day’s true step count. This error was remedied on the day it was identified.

### Daily Social Comparison Task

#### Overview

As in several previous studies, opportunities to make social comparisons came through viewing profiles of individuals described as similar to the participant [42]. After completing the motivation assessment, participants in each study had the opportunity to select one or more profiles to view. These profiles described other individuals who had recently engaged in more or less PA than the participant to represent upward or downward comparison targets at a range of distances from the participant’s own recent PA behavior. Profile options included only minimal information, including only their username (eg, “dmf25”) and step total. Participants were able to click on multiple selections to learn additional information but could only select 1 profile to view in full.

#### Study 1

**Figure 1:**
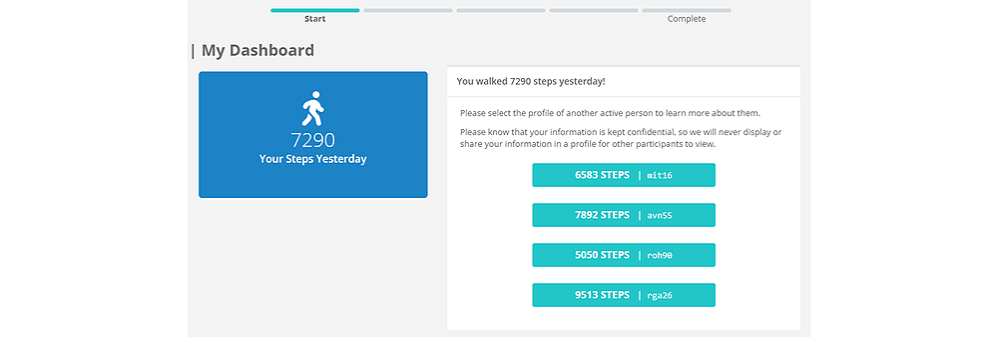
View of the study web page that included 4 comparison targets to select from.

Study 1 was designed as a proof-of-concept pilot to ensure that the systems worked correctly and that the platform could detect participants’ navigation behavior. Participants were asked to engage in a 5-minute session on the web platform once per day for 7 days. After logging in each day, participants were greeted with their own step total for the previous day, as tracked by their Fitbit device or app. This was posted next to 4 profiles of “other users,” which were created by the system; 2 presented upward comparisons (ie, with step totals of 110% and 130% of the participant’s steps from the day before), and 2 presented downward comparisons (ie, with step totals of 90% and 70% of the participant’s steps from the day before; Figure 1). In each case, a margin of –2% to +2% was applied as noise to protect against potential identification of the study’s aim.

**Figure 2:**
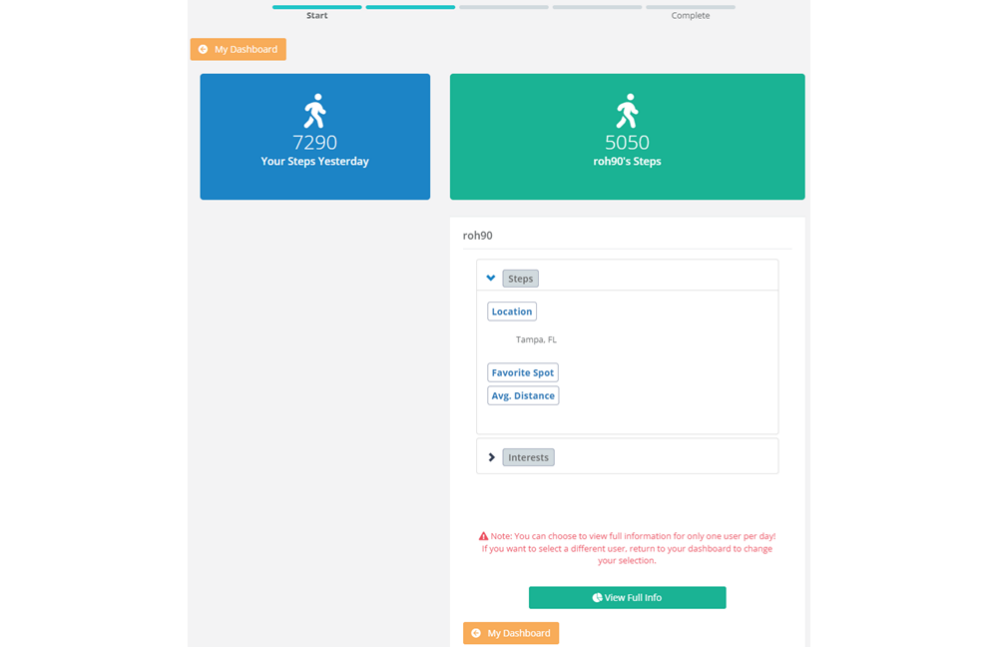
View of the Overview study web page, in which a profile has been initially selected but not yet selected to view in full. Participants could still go back and peruse other profiles to select from before selecting their final profile for full details (comparison target). Avg: average.

**Figure 3:**
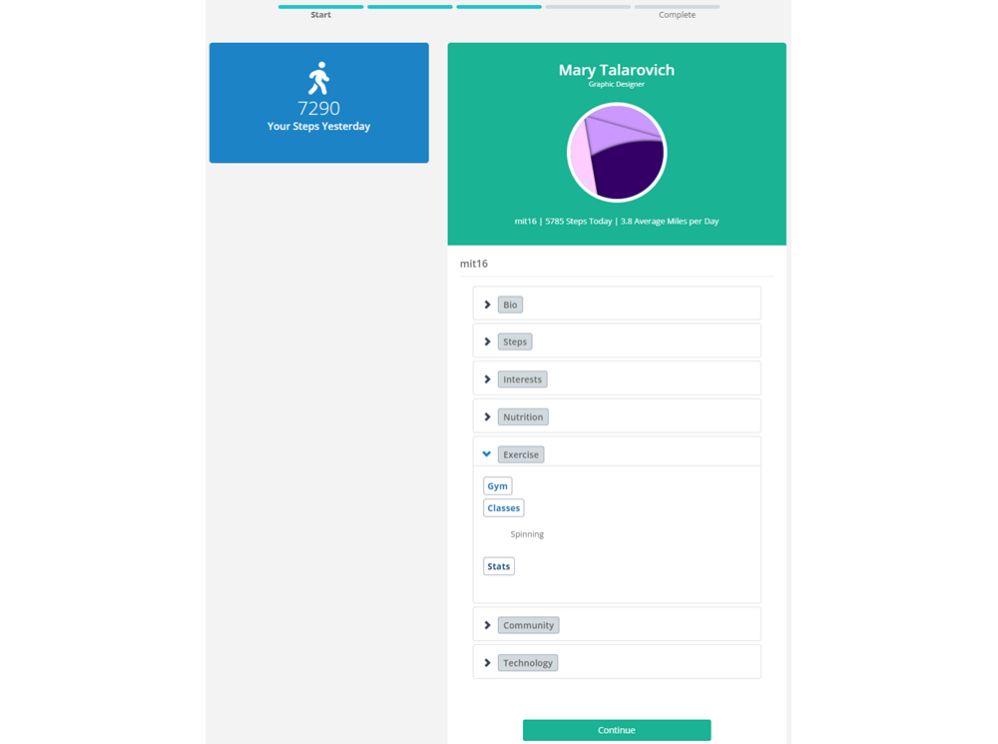
Once committing to a profile during a daily session, participants are taken to a Details page that lists full information regarding the profile.

As noted, participants could select multiple profiles to learn additional information about the users, including their city of residence and favorite location to exercise (as shown in Figure 2). However, they would have to select 1 profile to view in full to complete the task for the day. Upon selecting a profile to view in full, participants viewed a page containing a user’s demographics (eg, age, sex, and profession), physical appearance (eg, height and weight), exercise preferences (eg, preferred forms of PA), and other personal information (eg, hobbies; Figure 3).

#### Study 2

The goal of study 2 was to examine patterns of user profile selection (ie, comparison targets) and response with respect to PA motivation and behavior. A revised web platform facilitated engagement in a daily, 2-minute task involving the selection of potential social comparison targets (9 days total). After logging in each day, participants viewed a page displaying their step count from the previous day (as collected from the Fitbit API either via a Fitbit wearable device or a smartphone step tracker synced to the Fitbit app). After reporting their initial motivation to exercise (1-5 rating scale), participants were presented with 4 profiles of other “users” of the application, as in study 1. However, instead of offering a consistent set of profiles with respect to step total (ie, 70%, 90%, 110%, and 130% of the participant’s own steps), participants were assigned to one of the following profile sets each day: (1) all 4 profiles lower than the participant’s (downward options only) at 90%, 80%, 70%, and 60% of the participant’s own step total from the previous day; (2) a mix of profiles—2 downward (lower than the participant’s own step total from the previous day at 90% and 80%) and 2 upward (higher than the participant’s own step total from the previous day at 110% and 120%); and (3) all 4 profiles higher than the participant’s (upward options only) at 110%, 120%, 130%, and 140% of the participant’s own step total from the previous day.

In each case, a margin of –2% to +2% was applied as noise to protect against potential identification of the study’s aim. After viewing their selected full profile, participants were asked to report their exercise motivation a second time (1-5 rating scale).

#### Study 3

**Figure 4:**
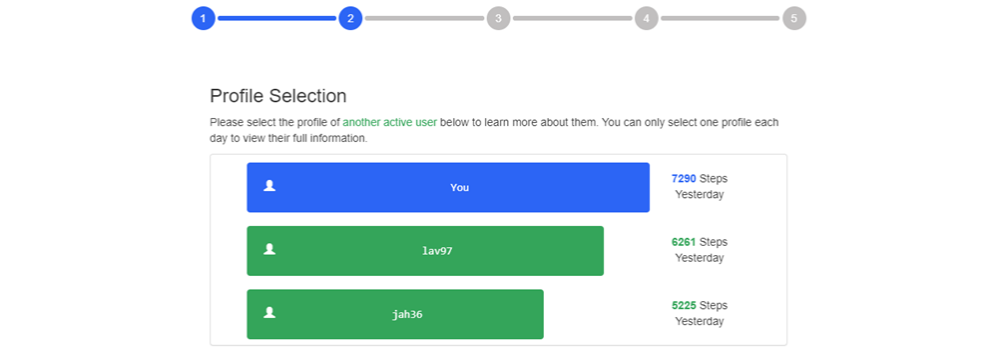
Options for selecting from 2 user profiles, listing them and the user in descending order and representing their step totals visually (ie, a leaderboard format).

The purpose of study 3 was to examine the translation of the profile selection platform to a gamified context, whereby participants were assigned to teams of 3 users. A further revised version of the web application allowed participants to view other users’ PA behavior and personal information (representing comparison targets) using a new format. As in study 2, participants were asked to log in and report their initial exercise motivation (1-5 rating scale). They then viewed brief descriptions of 2 additional profiles (as opposed to 4 in studies 1 and 2) in leaderboard format and were asked to select 1 to view additional information (Figure 4).

**Figure 5:**
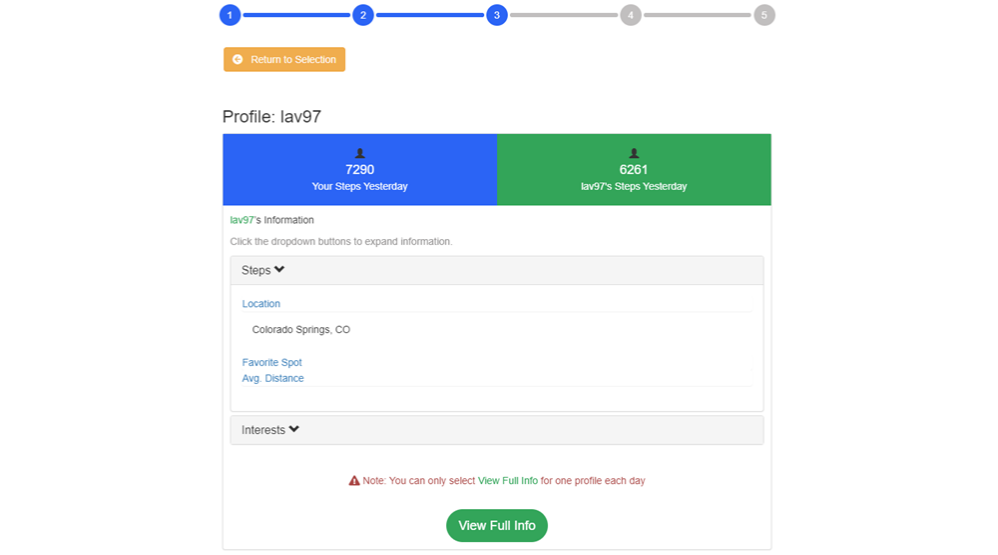
Initial profile view in study 3. Avg: average.

After selecting a profile, participants could view a subset of personal information (Figure 5); this view retained their own step total from the previous day to facilitate comparison with the selected user. Participants could access a full *Details* page once they selected a final profile to view in full.

However, unlike in the previous studies, step totals for other users in study 3 included data from other participants completing their data collection at the same time (ie, user data that were not created by the platform). Each participant was randomly assigned to a team with another user who began the study at the same time; these participants each saw the other’s step totals as 1 of their 2 profile options. The third user profile displayed in each session was generated and assigned by the platform, selected from the following options: (1) the third profile showed a step total 20% lower than the lower of the 2 live participants, and the individual participant had either the most steps or was in the middle; (2) the third profile showed a step total between that of the 2 live participants, and the individual participant had either the most or the least steps; and (3) the third profile showed a step total 20% higher than the higher of the 2 live participants, and the individual participant had either the least steps or was in the middle.

In each case, a random noise factor of –2% to +2% was added to obscure our process. This approach was designed to test manipulations of the game environment for the 2 live participant teammates by showing a fabricated third user who might provide an optimal comparison experience for the live teammates.

Across the studies, the distances between the user’s steps and the target’s steps (eg, 80% and 140%) were guided by the principle of offering a realistic range of options and by relevant literature. Specifically, there is evidence supporting the Köhler effect and “motivation gain” in a team game environment that shows that participants’ performance improves with a teammate who performs approximately 20% better than they do [43,44]. Under conditions in which users in this study saw both upward and downward targets as options, −20% was offered for symmetry. Other options were selected to retain realism while capturing distances from the user’s own steps that would be perceptible and large enough to show differences in associations with motivation or behavior. In study 3, the design particulars (ie, percentages below, between, or above 2 real users) resulted in a larger range and set of targets. A summary of each study design is presented in Table 2.

**Table 2:** Summary of design distinctions between the studies in this series (k=3).

Design feature	Study 1	Study 2	Study 3
Presentation format	Unordered list; user’s own steps from the day before next to list of target options	Unordered list; user’s own steps from the day before next to list of target options	Leaderboard (user ranked in descending order against 2 others)
Comparison target options presented each day, n	4	4	2
Range of comparison target distance from the user (steps per day), %	70-130	60-140	0->2000
Daily condition assignments	None—same set of target options presented each day (2 upward and 2 downward); step totals differed based on user’s own steps per day	Randomized to 1 of 3 sets of targets—upward targets only, downward targets only, or mixed (2 upward and 2 downward); step totals differed based on user’s own steps per day	Randomized to 1 of 3 sets of targets—upward targets only, downward targets only, or mixed (1 upward and 1 downward)

### Statistical Analyses

All analyses were conducted using SAS (version 9.4; SAS Institute). Missing data were minimal; data were missing for 20% (7/35) of days in study 1 (because of low compliance from 1 participant), 1% (5/477) of days in study 2, and 4.9% (24/486) of days in study 3. Additional data were removed from relevant analyses where unreasonable values were observed, including values for time spent viewing profiles (>6 minutes; 4 observations) and steps per day (<100; 38 observations). The resulting data sets for studies 2 and 3 included 472 and 387 observations, respectively. These data sets afforded power of >0.80 for the primary, within-person tests described in this section (*α* of .05 [45]), although we emphasize effect sizes throughout—PA is described in steps per day, and all other associations are described using semipartial correlation coefficients (*sr*). Between-person tests were included to describe potential trends only as power was limited by modest sample sizes.

We first used empty models to calculate intraclass correlation coefficients (ICCs) to determine the proportion of variance attributable to between-person stability in the outcomes of interest. This included participant navigation behavior when interacting with comparison target profiles (time spent viewing the selected profile and number of elements viewed) and PA outcomes (motivation to exercise and steps per day), which were treated as continuous in all models. Motivation was not assessed in study 1; total steps per day were assessed in all 3 studies. Change in motivation in studies 2 and 3 was calculated by subtracting motivation before profile selection from motivation after selection.

Our first aim was to describe PA-based comparison selections, including participant navigation of the platform and the comparison direction and scale of the selected profile. To address this aim, we initially examined whether gender, racial/ethnic identification, and age (age treated as continuous and centered at the grand mean) differentially predicted navigation behavior. We then used descriptives to examine the frequencies of user profile selections in categories, representing the user’s steps as a percentage of the participant’s steps from the previous day (rounded to the nearest 10). The direction and scale of comparison targets (profiles) selected (all studies), the direction or directions of targets presented using randomization (studies 2 and 3), and whether the selected profile represented the other active participant or the fabricated user (study 3) were treated as categorical and subsequently used as predictors of PA outcomes.

Our second aim was to examine day-level associations between comparison selections and PA outcomes (motivation to exercise and steps per day). Analyses used multilevel modeling techniques using SAS PROC MIXED with restricted maximum likelihood estimation to address the nested data structure (ie, days nested within individuals). Gender, racial/ethnic identification, and age were used as covariates in all multilevel models (studies 2 and 3), with comparison target direction and scale (all studies), the randomized set of targets (studies 2 and 3), and fabricated user versus not (study 3) as predictors of PA outcomes. Although users accessed the platform at a range of times across the days of observation in each study, sensitivity analyses showed that the time of day at which users accessed the platform was not associated with any of our outcomes of interest and did not meaningfully change the results or conclusions reported in the next section. For parsimony, we reported the results of all tests without time of day as an additional covariate.

Finally, new navigation behavior and motivation variables were created for studies 2 and 3: between- and within-person variance were distinguished by calculating each person’s mean across days (between-person) and the difference between this person’s mean and the response on a given day (within-person; ie, person-mean centering [46]). This allowed for testing whether steps per day were associated with within-person fluctuation in navigation behavior or motivation, controlling for typical navigation behavior or typical change in motivation from before to after comparison.

### Ethics Approval

All procedures were approved by the institutional review board of Drexel University (approval 1901006917).

### Informed Consent and Compensation

All participants provided documentation of informed consent. Compensation for participation was provided through either extra credit in college courses or electronic gift cards depending on individual preference.

## Results

### Study 1

Of the 5 individuals who participated in the initial proof-of-concept test, 4 (80%) completed the expected daily uses of the web platform (ie, 6-7 within 19 days of enrollment); 1 (20%) participant completed 2 daily uses during the allotted time frame. Participants elected to view the full profile for the first user they selected on 71% (20/28 selections) of days. Across days, participants spent an average of 40 (range 3.3-145) seconds on their selected full profile and clicked on an average of 5 (range 0-29) profile elements. Less than 40% of the variability in both the amount of time each participant spent on their selected profiles and the number of elements they elected to view was attributable to stable, between-person differences (ICC=0.28 and 0.36, respectively), suggesting considerable within-person variability in these behaviors across days (*P*<.001 in all cases).

Selecting to view the full profile of upward comparison targets was considerably more frequent than selecting downward targets, with upward targets representing 75% (21/28) of the observed selections. The most popular selection was the user with 130% of the participant’s own steps from the previous day (13/28, 46% of selections; Table 2). Relative to all other choices, participants spent slightly longer viewing targets with 110% of their own steps from the previous day (contrast *B*=18.53, SE 12.51 seconds; *F*_6_=2.19; *P*=.19) but clicked on more profile elements when viewing targets with 90% of their own steps from the previous day (contrast *B*=8.18, SE 3.31 clicks; *F*_6_=2.47; *P*=.05). Within-person, neither the amount of time spent viewing profiles nor the number of profile elements viewed were associated with steps per day (*P*=.53, *P*=.99 respectively). However, participants took nearly 4000 more steps on days when they selected upward targets than on days when they selected downward targets (*F*_1,3_=5.31; *P*=.10), with the most steps occurring on days when they selected targets with 110% of their own steps from the previous day (Table 3).

**Table 3:** Steps per day by profile (comparison target) selection; percentages represent the step totals of the selected profile relative to the participant’s steps from the previous day rounded to the nearest 10% (n=28).

Type of target	Frequency, n (%)	Steps per day, *B* (SE)
70%	3 (11)	4023.82 (2927.76)
90%	4 (14)	1448.51 (2665.40)
110%	8 (29)	7152.59 (2321.05)
130%	14 (50)	6081.24 (2050.10)
Downward (70% or 90%)	7 (25)	2241.73 (2358.08)
Upward (110% or 130%)	21 (75)	6403.27 (2015.87)

### Study 2

Similar to study 1, participants elected to view the full profile for the first user they selected on the vast majority of days (425/472, 90% of selections). Across days, participants spent an average of 18 (range 1.4-130) seconds on their selected full profile and clicked on an average of 9 (range 0-64) profile elements. Most of the variability in both the amount of time each participant spent with their selected profiles and the number of elements they elected to view was attributable to stable, between-person differences (ICC=0.53 and 0.63, respectively), although both showed evidence of fluctuation for the same person across days (*P*<.001 for both within-person variance components). Men spent slightly longer viewing each profile and selected to view more profile elements than women (*P*=.09 and *P*=.13, respectively); both behaviors were also positively associated with age (*P*=.02 and *P*=.02, respectively). However, neither time spent viewing nor the number of elements selected meaningfully differed based on racial/ethnic identification, the set of profile options presented, or the type of target selected (*P*=.63, *P*=.11, *P*=.39, *P*=.36, *P*=.91, *P*=.56, respectively).

Upward comparison target selections were slightly more frequent than downward comparison target selections, representing 54.2% (258/476) of all final profile selections. However, overall, the most popular comparison target selection for viewing the full profile were downward targets at 90% of the participant’s steps from the previous day (Table 4). On days when only downward target options were presented, participants most often selected the target with the step count closest to their own (ie, 90% of their steps from the previous day); this trend was reversed on days when only upward target options were presented (ie, 140% of their steps from the previous day, the farthest from their own). When presented with both upward and downward target options, they selected the target with the highest overall step count (ie, 120% of their steps from the previous day).

Average change in motivation from before to after selection was slightly positive across the days (*B*=0.10, SE 0.05), with considerable within-person variability (ICC=0.18). The lowest increases in motivation occurred on days when only downward target options were presented (Table 4). Interestingly, participants showed *decreases* in motivation to exercise only on days when they selected targets with 60% and 110% of their own steps from the previous day (Table 4). These represented the farthest downward and closest upward targets from their own steps, respectively. Participants showed increases in motivation on days when they selected all other targets (contrast *F*_409_=5.38; *P*=.02; *sr*=0.32), and this trend did not change when controlling for the set of target options shown.

With respect to steps per day, participants took approximately 540 *fewer* steps on days when both downward and upward target selections were presented relative to only upward or only downward targets (contrast *F*_417_=−3.80; *P*=.05). Steps were highest on days when participants selected targets most distant from themselves in both directions—they took approximately 725 more steps on days when they selected targets with 60% and 140% of their own steps from the previous day relative to targets closer to their own steps (contrast *F*_409_=3.76; *P*=.05). As noted, participants did not always select targets that led to increases in motivation to exercise. Within-person, neither motivation nor steps differed based on the amount of time spent viewing the selected profile or the number of profile elements viewed (*P*=.28, *P*=.21, *P*=.81, *P*=.90, respectively). However, controlling for their typical change in motivation to exercise from before to after comparison, on days when participants were more (vs less) motivated than usual after viewing their selected target, they engaged in more steps (*F*_1,418_=9.24; *P*=.003).

**Table 4:** Motivation to exercise and steps per day by profile (comparison target) selection in study 2; percentages represent the step totals of the selected profile relative to the participant’s steps from the previous day rounded to the nearest 10% (n=472).

		Frequency, n (%)	Change in motivation to exercise, *B* (SE)	Steps per day, *B* (SE)
**Type of target selected**				
	60%	34 (7.2)	−0.04 (0.11)	6932.36 (597.06)
	70%	32 (7.2)	0.11 (0.11)	6215.89 (605.29)
	80%	51 (10.8)	0.21 (0.09)	5697.40 (515.19)
	90%	100 (21.2)	0.02 (0.07)	6356.75 (418.57)
	110%	84 (17.8)	−0.01 (0.08)	6078.63 (444.95)
	120%	93 (19.7)	0.14 (0.07)	6447.72 (424.89)
	130%	21 (4.4)	0.20 (0.14)	6515.59 (733.75)
	140%	60 (12.7)	0.19 (0.09)	6965.72 (733.75)
**Type or types of target options shown**				
	Downward only	159 (33.7)	0.04 (0.06)	6573.20 (367.93)
	Downward and upward (2 each)	159 (33.7)	0.12 (0.06)	6020.79 (366.82)
	Upward only	159 (33.7)	0.11 (0.06)	6556.29 (368.49)

### Study 3

Participants elected to view the full profile for the first user they selected on 96.9% (375/387 selections) of occasions. Across days, participants spent an average of 72 (range 1-351) seconds on their selected full profile and clicked to view an average of 12 (range 0-54) profile elements. As in study 2, although the amount of time each participant spent with their selected profiles and the number of elements they elected to view were fairly stable (ICC=0.58 and 0.65, respectively), they showed some variation for the same person across days (within-person variance components; *P*<.001 in all cases). The time spent viewing profiles and the number of profile elements selected were again positively associated with age (*P*=.04 and *P*=.03, respectively), although neither behavior was associated with the set of profile options presented, whether the selected profile represented an upward or downward target, or whether the selected profile was of the fabricated user versus the real participant (*P*=.63, *P*=.75, *P*=.88, *P*=.92, *P*=.14, *P*=.80, respectively). However, unlike in study 2, neither the amount of time spent on the selected profile nor the number of profile elements selected differed by gender or racial/ethnic identification (*P*=.93, *P*=.34, *P*=.93, *P*=.35, respectively).

The method used to generate profiles in study 3 resulted in participant selections of comparison targets ranging from 0% to 20,610% of their steps from the previous day. This represented selections of users with step totals ranging from 0 to 21,132 steps, with 88 selections of users who had <1000 steps and 27 selections of users with >10,000 steps. This generated >90 individual categories of selection, with most of these categories representing upward targets (ie, the selected users had more steps than the participants on the previous day). For ease of interpretation, upward selections were recategorized by percentages of the participants’ steps, as shown in Table 5. Participants selected the fabricated user on most days (210/387, 54.3%); they were more likely to choose the fabricated user when they selected upward (vs downward) targets (*F*_1,336_=4.44; *P*=.04) and were least likely to choose the fabricated user when that user was shown as last on the leaderboard (*F*_2,335_=10.20; *P*<.001).

As in studies 1 and 2, upward selections were more frequent than downward selections and represented 57.1% (221/387) of all targets selected. However, unlike in study 2, the most popular choice overall was upward at 120% of the participants’ steps from the previous day (55/387, 14.2% of selections; Table 5). Users with 120% of the participants’ steps from the previous day represented 41.4% (53/128) of all selections on days when the fabricated participant was at the top of the leaderboard but <1% (1/127, 0.8% and 1/132, 0.8%) of selections on days when the fabricated user was second or third. Close in overall frequency of selections were users with 80% of the participant’s steps (as in study 2; 41/387, 10.6% of selections) and users with 200% to 999% of the participant’s steps (41/387, 10.6% of selections). Of note, selecting to view the profile for a user with the same number of steps the participant had on the previous day occurred on 2.3% (9/387) of the days.

**Table 5:** Change in motivation to exercise before to after profile (comparison target) selection and steps per day by profile selection in study 3; percentages represent the step totals of the selected profile relative to the participant’s steps from the previous day rounded to the nearest 10% (n=387).

			Frequency, n (%)		Change in motivation to exercise, *B* (SE)		Steps per day, *B* (SE)
**Type of target**							
	0%	10 (2.6)		−0.07 (0.27)		3838.82 (1160.46)	
	10%	8 (2.1)		−0.09 (0.30)		2635.07 (1137.18)	
	20%	13 (3.4)		0.06 (0.24)		3964.32 (922.67)	
	30%	6 (1.6)		0.18 (0.35)		3697.40 (1277.26)	
	40%	5 (1.3)		−0.74 (0.38)		3263.22 (1391.01)	
	50%	21 (5.4)		0.24 (0.20)		3004.35 (874.38)	
	60%	21 (5.4)		0.39 (0.20)		3404.35 (1127.69)	
	70%	19 (4.9)		0.50 (0.21)		3221.15 (1130.97)	
	80%	41 (10.6)		−0.03 (0.16)		2243.21 (901.90)	
	90%	22 (5.7)		0.10 (0.20)		3440.08 (877.97)	
	100%	9 (2.3)		0.32 (0.29)		3337.31 (1432.54)	
	110%	14 (3.6)		0.07 (0.24)		3598.14 (1053.33)	
	120%	55 (14.2)		−0.09 (0.14)		3454.85 (747.45)	
	130%	9 (2.3)		0.28 (0.29)		3221.15 (1130.97)	
	140%	11 (2.8)		−0.11 (0.26)		2422.84 (1127.69)	
	150%	4 (1)		0.16 (0.42)		3448.54 (1551.14)	
	160%	6 (1.6)		−0.25 (0.35)		3891.26 (1299.39)	
	170%	10 (2.6)		0.06 (0.27)		3345.57 (1093.40)	
	180%	5 (1.3)		0.14 (0.38)		5536.70 (1382.12)	
	190%	6 (1.6)		0.51 (0.35)		1878.49 (1378.49)	
	200%	5 (1.3)		0.04 (0.38)		2900.83 (1372.52)	
	110%-199%	18 (4.7)		−0.12 (0.21)		3675.08 (938.78)	
	200%-999%	41 (10.6)		0.29 (0.16)		2949.21 (781.46)	
	1000%-1999%	8 (2.1)		−0.10 (0.31)		3915.49 (1299.92)	
	>2000%	20 (5.2)		−0.16 (0.21)		3771.33 (962.49)	
**Type or types of target options shown**							
	Participant either first or second on leaderboard (fabricated user was third or last)	127 (32.8)		0.05 (0.11)		3510.65 (667.93)	
	Participant either first or third (last) on leaderboard (fabricated user was second)	132 (34.1)		0.17 (0.11)		3033.56 (669.24)	
	Participant either second or third (last) on leaderboard (fabricated user was first)	128 (33.1)		0.02 (0.11)		3573.50 (668.77)	
**Selected fabricated user**							
	No	177 (45.7)		0.06 (0.11)		3248.29 (653.82)	
	Yes	210 (54.3)		0.09 (0.10)		3463.86 (642.90)	

Average change in motivation to exercise from before to after selection was again positive across days but extremely small (*B*=0.08, SE 0.51), although within-person variability was predominant (ICC=0.04). Increases in motivation were largest on days when participants selected users with 190% of their steps from the previous day, followed by users with 70% of their steps from the previous day (Table 5). Participants’ motivation *decreased* on days when they selected upward targets with steps farthest from their own (ie, >2000% of their steps from the previous day) as well as on days when they selected users with 10%, 40%, 80%, 120%, and 160% of their steps from the previous day; the greatest decreases were seen on days with selections of 40% of the participants’ own steps from the previous day. Change in motivation was highest on days when the fabricated user was placed between a given participant and the other real participant on the leaderboard relative to days when the fabricated user appeared above or below both real participants (contrast *F*_335_=2.34; *P*=.12; *sr*=0.17). Change in motivation did not meaningfully differ between days when participants selected an upward or downward target (collapsed across percentage categories; *F*_47_=.97; *P*=.34) or between days when they selected the fabricated user versus the other live participant (*F*_46_=.00; *P*=.98).

With respect to steps per day, participants took approximately 500 *fewer* steps on days when the fabricated user was placed between themselves and the other real participant on the leaderboard relative to days when the fabricated user appeared above or below both real participants (contrast *F*_303_=2.89; *P*=.09). Steps did not meaningfully differ between days when participants did and did not select to view the profile of the fabricated user (*F*_46_=.56; *P*=.46). Although steps also did not differ overall based on the comparison direction and scale of the selected profile (*P*=.90, *P*=.99, respectively), interestingly, steps were highest on days when participants selected users with 180% of their own steps from the previous day (approximately 5500 steps) and lowest on days when they selected users with 190% of their own steps from the previous day (approximately 1900 steps; Table 5). Steps also did not meaningfully differ between days when participants selected an upward versus a downward target (collapsed across percentage categories; *P*=.90).

Neither motivation nor steps were associated with daily fluctuation in the amount of time each participant spent on their selected profiles or the number of elements they elected to view (within-person; *P*=.60, *P*=.64, *P*=.38, *P*=.34, respectively). Finally, although the within-person association between participants’ motivation and steps per day was not significant (*F*_304_=1.11; *P*=.29), it was noteworthy that the direction of the association was negative—unlike in study 2, on days when they were more motivated than usual after viewing their selected profile, participants took *fewer* steps than usual (*B*=−186.84, SE 177.65).

## Discussion

### Principal Findings

Social comparison processes can be activated to promote PA in digital environments, although individuals’ interactions with and responses to self-selected comparison targets in this context are poorly understood. As social comparison features are already built into many existing digital PA tools [14,16,23], this series of studies was designed to provide additional information about this important aspect of digital PA promotion. We created unique web-based platforms to capture individuals’ selections of social comparison targets, their interactions with information about the selected targets, and their subjective responses to the selected targets over 7 to 9 days, as well as their PA behavior on each of these days. We observed several similarities and differences across these studies that can shed additional light on this area.

First, participants chose to view the full profile of the first participant they selected on the vast majority of days (71%-97%), although many participants explored other profiles before returning to and settling on the first one they had selected. Participants also interacted with the platform and their selected profiles differently across days. They did not merely settle into a pattern of the same behavior each day despite the consistency and simplicity of the task. This underscores the appeal of PA-based comparisons and their potential to sustain engagement with digital tools, although additional testing over longer periods is needed.

Second, in both studies where demographic information was collected, older participants spent more time viewing profiles and selected more profile elements to view than younger participants. This stands in contrast to existing cross-sectional evidence, which suggests that older people are less interested in comparisons than younger people [47]. It is possible that our findings reflect a general tendency among older people to pay more attention to their participation in research than younger people [48]. Alternatively, it is possible that cross-sectional, retrospective self-evaluations of comparison activity do not align with observable behavior; this potential discrepancy is worthy of further investigation given that social comparison is often captured using global self-report measures [49,50]. Also noteworthy is that, although the participants’ ages in these studies ranged from 18 to 56 years, we recruited students enrolled in college who were predominantly in their early 20s. As such, associations with age warrant further investigation. Other observations of differences in behavioral interactions with social comparison information based on demographics (eg, gender) were not consistent across the studies in this series, although the power for these comparisons was limited.

Third, across all studies, the profiles of upward comparison targets were selected for full viewing more often than those of downward comparison targets. This was not an artifact of randomized exposure—each participant had an equal number of opportunities to select upward and downward targets. Moreover, participants tended to select upward targets that were distant from themselves (ie, those who had many more steps than they had) rather than upward targets closer to themselves. Selecting to make upward comparisons, particularly when a range of options is available, is often motivated by a desire for self-improvement [51,52]. Given that participants in these studies indicated that PA is important to them, selecting targets doing extremely well with PA offered an opportunity to learn information from that target that could support achievement of a similar high status [53]. For example, participants could learn new ways to be active from the profiles of very active participants, giving them opportunities to set PA goals to model the target.

However, despite the relative popularity of upward targets, participants also frequently selected downward targets and tended to select downward targets close in steps to their own (vs more distant from their own). Self-selection of downward targets is often motivated by a desire for self-enhancement [51,52]; seeing oneself as doing better than someone else in a valued domain can be satisfying and provide an emotional boost. The variety of selections across days may indicate day-to-day variability in participants’ needs and immediate goals that could be met with comparison opportunities [54,55].

Importantly, participants did not always select the target that was most useful with respect to either subjective PA motivation or PA behavior—many selections were associated with decreases in motivation, low PA engagement, or both. Similarly, a participant’s change in PA motivation as a result of viewing their selected comparison target was not consistently associated with their PA behavior. Subsets of previous work in this area show important aspects of comparisons that may help explain these findings and, thus, warrant further consideration. One is that people do not always select the comparison opportunities that fulfill either self-improvement or self-enhancement goals; at times, their intentions are to confirm that their own situation is bad or could worsen or to justify not making difficult behavior changes such as increasing their PA (eg, “I’m already doing better than someone else, so I’m doing fine” [56,57]). Even when they do have positive, goal-oriented intentions for selecting particular comparison opportunities (eg, to learn important information or to feel better), their expectations are not always met by the target provided [58]. In such situations, the comparison opportunity may actually lead to negative outcomes.

In addition, the affective consequences and behavioral correlates of a social comparison selection opportunity may depend on how the comparer interprets the information they receive. The Identification-Contrast Model of comparison processes [59] proposes that the comparer can focus on either similarities or differences between themselves and a target (reflecting identification with vs contrast against the target, respectively). Identifying with an upward target highlights the possibility that the comparer can achieve similar (better) outcomes, and contrasting against a downward target highlights the comparer’s current success (as the outcome could be worse). Conversely, identifying with a downward target suggests that the comparer’s situation is bad or may become worse; contrasting against an upward target highlights the comparer’s inferiority and suggests that the likelihood of achieving similar success is low. In the context of PA and similar comparisons of health behaviors, there is recent evidence showing that greater (vs less) identification with active others is associated with more frequent attendance to exercise classes [60], and identification and contrast processes moderate the association between the type of target selected (upward vs downward) and motivation to engage in healthy behavior [28]. Identification and contrast with respect to both upward and downward comparisons are also known to differ between people and show evidence of fluctuation for the same person over time [61-63]. Thus, in this series of studies, the high day-to-day variability in participants’ PA outcomes that were not fully explained by the direction or scale of the selected target may be due to individual or day-level differences in the extent of identification or contrast with the target. Assessment of these processes in future work could more fully explicate the complexity of social comparison and its optimal use to promote PA engagement. As discussed further in this section, to effectively isolate the source of this variability, removing potential noise coming from variability in the time of day of social comparison selections and exposure would be optimal in future studies.

Finally, we observed differences in findings between studies that may generate additional hypotheses to be tested in future work. For example, PA motivation in response to viewing the selected comparison target was positively associated with within-person behavior in study 2 but not in study 3. Study 2 presented the list of target selection options and the selected target’s step total side by side with the participant’s step total from the previous day. In contrast, study 3 presented social comparison target selection options in a leaderboard format such that the participant saw a visual representation of their rank against the 2 other users. These differences may affect the psychological dynamics of comparison selections and their associations with PA motivation and behavior, in general or for specific individuals. Target selection options in study 3 also included both a real participant and a fabricated user, where the ultimate goal was to determine the optimal placement of the fabricated user to balance the comparison effects on both of the real users. In this study, PA motivation increased the most on days when the fabricated user was in the middle of the leaderboard (between the 2 real users), but steps were lowest on these days. The leaderboard and balance approach may have blunted the potential negative effects of comparisons but also blunted some positive effects.

Participants who enrolled in study 2 were also noticeably more active than those who enrolled in study 3 (and study 1); relative to the US guideline of achieving 10,000 steps per day [6], the average activity level was moderate in study 2 and low in study 3 (and study 1). It is possible that the general correspondence between PA motivation and behavior is stronger for those who are moderately active than for those who are inactive in that those who are moderately active are better able to enact their PA motivation. Distinctions between studies could be due to participant characteristics, study design, or a combination of both. As a result, it is not yet clear whether one study design is more useful than another for activating beneficial PA-based social comparisons or whether there is a subset for whom one is superior to another.

### Strengths and Limitations of This Research

This series of studies has several strengths. Specifically, all 3 studies used objective assessment of comparison target (profile) selection, interactions with the target (ie, time spent viewing and number of profile elements viewed), and PA behavior (steps per day) across several days. Studies 2 and 3 also captured motivation to exercise both before and after target selection using a momentary item that was tested in previous work [28,38]. Retention of enrolled participants was high across studies, with minimal missing data. In addition, we used a multilevel analytic approach that allowed for maximizing the utility of intensive repeated assessments, with insights into daily behavior across participants as well as within-person associations across days. Finally, we took an iterative approach such that the platforms used in each study were slightly different with respect to the comparison target options to allow for preliminary comparisons between and across studies. Although the sample sizes in each study were modest and do not afford definitive conclusions about the sources of divergent results, observations of consistency and inconsistency across studies provide a strong foundation for hypothesis-driven research on a larger scale.

In addition to modest sample sizes, several other limitations are noteworthy. Participants’ access to the web platform was not restricted to a particular time of day or constrained to be consistent for the same participant across days. Consequently, participants may have taken part at varying times of day (eg, before vs midway through vs after engaging in most of their steps for that day). Although participants’ comparisons were anchored to their steps for the previous day, which were already completed, and controlling for time of day did not alter our findings, this inconsistency could mask any effects of social comparison selections on motivation or PA behavior for the current day by allowing for considerable noise between and within participants. In addition, the precision of PA behavior captured likely varied by participant as some used wearable PA monitors (eg, Fitbit wristbands) whereas others used less sensitive smartphone accelerometers. Assessment of PA motivation and behavior was also misaligned—motivation referred to “exercise” (ie, structured bouts of sustained, moderate– to vigorous–intensity movement), and behavior was captured with respect to steps (ie, overall movement at any intensity, including light activity). Although motivation did predict within-person behavior in study 2, this discrepancy may further help explain the lack of association in study 3. Future work should ensure that assessments of cognitive determinants of PA and PA behavior refer to the same behavioral outcomes.

Finally, participants were all students enrolled in college courses who reported that PA was important to them. This ensured that the dimension of comparison (PA) was relevant to the participants [15]. The average participant in each study also fell far short of US recommendations for PA behavior (ie, 10,000 steps per day), suggesting that participants generally represented individuals who could benefit from increasing PA—a target population of interest. However, recruitment from college courses and requiring participants to endorse a preexisting interest in PA resulted in samples of well-educated, motivated, and predominantly White young adults. As noted, there is existing evidence indicating that younger adults report more interest in and show stronger responses to social comparison information than older adults [47]. This may limit the effectiveness of social comparison processes as a PA promotion tool for younger adults, who already tend to be more active than older adults in the United States [11]. These are common problems in digital health research, particularly early-stage work. Additional attention needs to be paid to recruiting and retaining diverse samples to fully understand the range of PA social comparison preferences and responses that may be useful for promoting PA.

### Conclusions

Despite these limitations, these findings have several important implications. With respect to platform interface design, users show interest in viewing the profiles of other users and engage with profile content when the initial information available offers social comparison opportunities. Furthermore, as social comparison target selections are often not associated with benefits for PA motivation or behavior, the current real-world conditions for digital PA promotion tools (which offer unrestricted access to other users [14]) do not appear to meet users’ needs. Outcomes could be improved with subtle manipulation of comparison target options. These exploratory findings show that constraining users’ PA-based social comparison options and changing their options across days (with respect to direction and scale) is both feasible and acceptable, with high completion rates. An important next step is to identify the people and immediate contexts for which certain types of comparisons are optimal (eg, older vs younger adults, men vs women, or high vs low precomparison motivation) to allow for systems to offer the PA-based social comparison opportunities that are most likely to benefit users in their daily lives.

## Abbreviations

API: application programming interface
ICC: intraclass correlation coefficient
PA: physical activity
sr: semipartial correlation coefficient
